# P-2014. A Quality Improvement Project to Reduce the Length of Stay for Patient Admitted with Osteomyelitis

**DOI:** 10.1093/ofid/ofaf695.2178

**Published:** 2026-01-11

**Authors:** Brian Grundy, Nipun Atri, Karrine Brade, Jeffrey Glasheen, Lakshmi Chauhan

**Affiliations:** University of Colorado, Aurora, Colorado; University of Colorado, Aurora, Colorado; University of Colorado Hospital, Aurora, Colorado; University of Colorado, Aurora, Colorado; University of Colorado, Aurora, Colorado

## Abstract

**Background:**

The diagnosis and management of patients with osteomyelitis, usually secondary to diabetic foot infections, is complex and requires a multidisciplinary approach for optimal management. We aimed to improve the care of patients with osteomyelitis through a multimodal group of interventions to optimize the value of care and reduce the length of stay for patients admitted with osteomyelitis.

**Figure:**
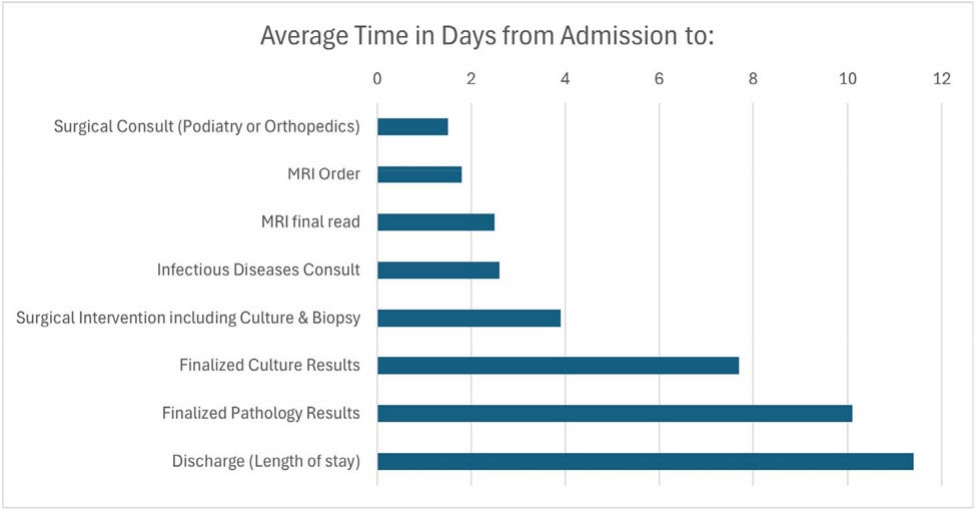
Average Cycle Times from Admission to Key Processes in the Diagnosis and Management of Osteomyelitis

**Figure:**
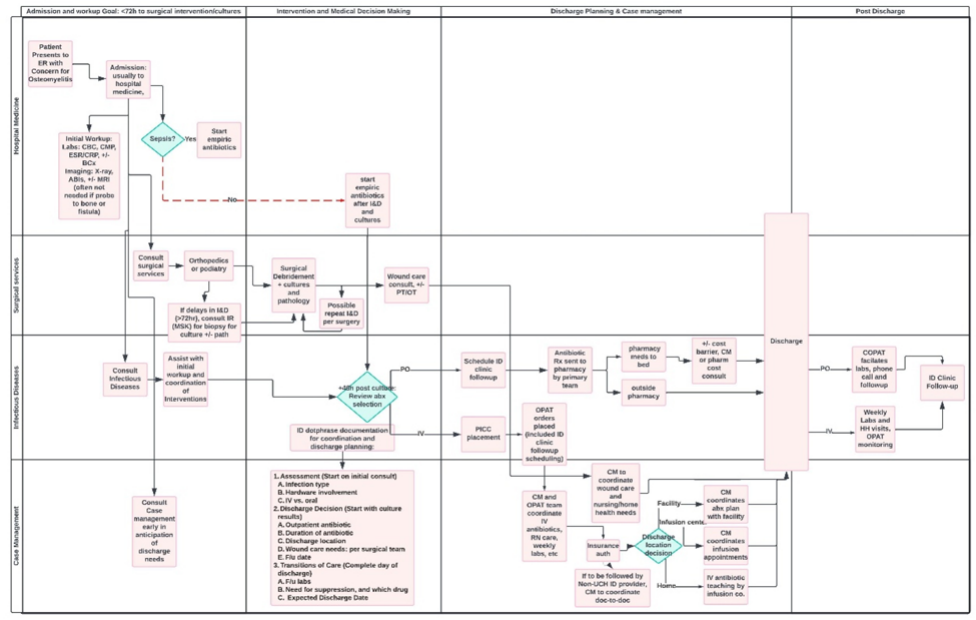
Simplified Ideal Process Map for Patients Admitted with Osteomyelitis Processes for a patient from admission through discharge and up to clinic followup, with many interventions to reduce length of stay and improve care for patients with osteomyelitis. Includes swim lanes during different time points during admission and beyond as well as different hospital services including hospital medicine, infectious diseases, surgery and case management.

**Methods:**

We evaluated patient records at a tertiary care hospital over 2 years from 2022-2023 for patients admitted with a diagnosis of osteomyelitis to better identify potential opportunities for improvement.

**Results:**

Over 2 years there were a total of 324 patients admitted with osteomyelitis, of which the average length of stay was 11.4 days and 39% of these patients were discharged on IV antibiotics. The average time from admission to ID consult and surgical consult was 2.6 days and 1.5 days, respectively. The average time from admission to MRI read and surgical intervention was 2.5 days and 3.9 days, respectively. The average time from admission to finalized culture and pathology results was 8 days and 10.1 days, respectively (Figure 1). The average time to ID clinic follow-up after discharge was 13.3 days.

**Conclusion:**

To reduce the hospital length of stay for patients with osteomyelitis, we identified several opportunities for improvement and planned several interventions. These included partnering with emergency and hospital medicine to improve the process for the initial diagnostic workup for osteomyelitis, including earlier ID consults, reducing unnecessary MRI use, reducing admissions chronic osteomyelitis and reducing time to initial surgical management (Figure 2). Additionally, ID consultants will not wait for pathology results prior to discharge and can follow up these results to guide management at a closer clinic follow-up visit. Additionally, we partnered with case management and developed a standardized note template to assist in discharge planning needs. We have also created a nurse navigator role in clinic to assist with patient monitoring for patients on PO antibiotics in addition to our already established OPAT program. These interventions have been implemented and will be monitored with the goal to reduce the length of stay for osteomyelitis by greater than 1 day over the next year.

**Disclosures:**

All Authors: No reported disclosures

